# Rare and common variant discovery by whole-genome sequencing of 101 Thoroughbred racehorses

**DOI:** 10.1038/s41598-021-95669-1

**Published:** 2021-08-06

**Authors:** Teruaki Tozaki, Aoi Ohnuma, Mio Kikuchi, Taichiro Ishige, Hironaga Kakoi, Kei-ichi Hirota, Kanichi Kusano, Shun-ichi Nagata

**Affiliations:** 1 Genetic Analysis Department, Laboratory of Racing Chemistry, 1731-2, Tsurutamachi, Utsunomiya, Tochigi 320-0851 Japan; 2 Equine Department, Japan Racing Association, 6-11-1, Roppongi, Minato, Tokyo, 106-8401 Japan

**Keywords:** Genetics, Zoology

## Abstract

The Thoroughbred breed was formed by crossing Oriental horse breeds and British native horses and is currently used in horseracing worldwide. In this study, we constructed a single-nucleotide variant (SNV) database using data from 101 Thoroughbred racehorses. Whole genome sequencing (WGS) revealed 11,570,312 and 602,756 SNVs in autosomal (1–31) and X chromosomes, respectively, yielding a total of 12,173,068 SNVs. About 6.9% of identified SNVs were rare variants observed only in one allele in 101 horses. The number of SNVs detected in individual horses ranged from 4.8 to 5.3 million. Individual horses had a maximum of 25,554 rare variants; several of these were functional variants, such as non-synonymous substitutions, start-gained, start-lost, stop-gained, and stop-lost variants. Therefore, these rare variants may affect differences in traits and phenotypes among individuals. When observing the distribution of rare variants among horses, one breeding stallion had a smaller number of rare variants compared to other horses, suggesting that the frequency of rare variants in the Japanese Thoroughbred population increases through breeding. In addition, our variant database may provide useful basic information for industrial applications, such as the detection of genetically modified racehorses in gene-doping control and pedigree-registration of racehorses using SNVs as markers.

## Introduction

The Thoroughbred breed was formed by crossing Oriental horse breeds and British native horses and has been selected by race for approximately 300 years^1^; these horses are currently used worldwide for horseracing. Although 88,441 Thoroughbreds were produced and registered worldwide in 2018^2^, the number of sires was only 6634. Sires are usually stallions selected for use in breeding programs based on their racing careers and evidence that they can pass on their performance capabilities to their offspring.

At maturity (3 years old), a Thoroughbred is approximately 163 cm in height and 470 kg in body weight^3^. These horses typically have delicate heads, slim bodies, broad chests, and short backs. In addition, they have both excellent speed and stamina for running racecourses from 1000 to 3000 m. The phenotypes of these traits may be associated with genetic information, and several causative genes and/or variants have already been identified by genome-wide association studies (GWAS) and targeted gene sequencing^4–8^. To identify causative genes and variants of several traits in Thoroughbred, detailed variant information of this breed should be obtained through genetic studies.

Another application of genomic data is to devise controls for gene doping. Doping control is an important factor in organised horseracing, and the International Federation of Horseracing Authorities (IFHA) has prohibited gene doping along with conventional doping^9,10^. Gene doping in horseracing can be divided into two categories: (1) administration of gene-doping substances to postnatal animals and (2) the generation of genetically modified racehorses. Although detection methods for the former have been developed^11–16^, no methods are available for detecting genetic modifications. For this purpose, whole-genome sequencing^17^, which requires a detailed understanding of variations of the Thoroughbred genome, may be useful.

The horse genome was first sequenced with a 2.33-Gb draft assembly (31 autosomal and X chromosomes) and was published in 2009 as EquCab2.0^18^. Sequence annotation by the ENSEMBL pipeline predicted 20,322 protein‐coding genes, which is comparable to that in humans, mice, and other mammals. The latest version of the horse genome is EquCab3.0^19^, with many gaps eliminated from EquCab2.0, resulting in a total read length of 2.41 Gb^20^.

In EquCab2.0, most of the genome of a Thoroughbred mare named Twilight, donated by Cornell University, was sequenced, after which several other breeds (Akhal-Teke, Andalusian, Arabian, Icelandic, American Quarter Horse, Standardbred, Belgian, Hanoverian, Hokkaido, and Fjord) were used to detect single-nucleotide variants (SNVs). This facilitated the cataloguing of over one million SNVs to compare genetic variation within and between different breeds^18^. Recently, the whole genomes of 88 horses from 25 breeds were sequenced by next-generation sequencing based on paired-end reads^21^. Approximately 23.5 million SNVs have been detected in horses. However, no studies have analysed a large number of individuals of a single breed to discover rare and common variants.

The purpose of this study was to clarify the genomic variations of a Thoroughbred population by performing whole-genome sequencing (WGS) of 101 unrelated Thoroughbreds and to construct a variant database for basic studies and industrial applications, such as disease control by identifying candidate variants for complex traits and gene-doping control.

## Results and discussion

### Detected SNVs

In this study, we used WGS data from 101 unrelated Thoroughbred horses born in Japan, or born in the USA, the UK, Ireland, or France and then imported to Japan. WGS data were obtained using Illumina paired-end (150 bp) sequencing technology with 36.8-fold coverage on average (range 29.5–54.2) (Table 1, Supplementary Table S1). It was expected that high coverage would lead to accurate SNV calling.

**Table 1:** Minimum, maximum, and mean numbers of mapped sequence reads and SNVs detected in 101 Thoroughbred racehorses.

	Minimum	Maximum	Mean
Mapped region (× 1)	2,485,336,485	2,490,187,301	2,488,579,272
Mapped region (× 10)	2,384,904,267	2,557,836,663	2,438,085,403
Mapped region (× 30)	896,891,559	2,214,548,786	1,495,177,119
Coverage	29.5	54.2	36.8
Detected SNVs	4,848,226	5,343,721	5,122,752
Filtered SNPs	4,432,698	4,865,479	4,656,698

**Figure 1:**
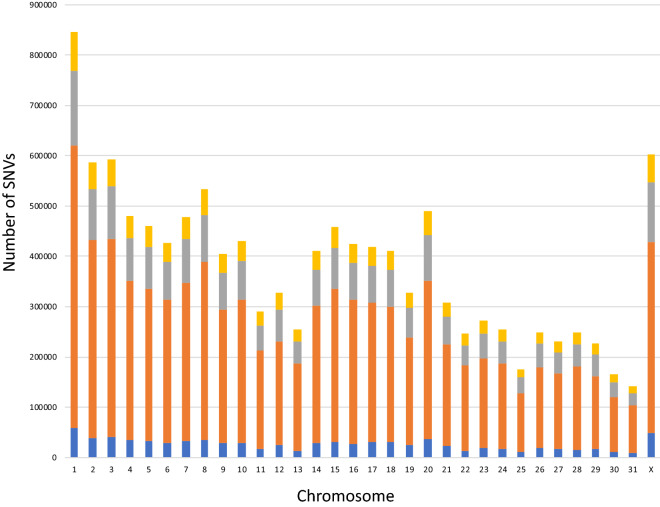
Distribution of single-nucleotide variants (SNVs) detected in 101 Thoroughbred horses. Blue: A to T or T to A; orange: A to G (T to C) or G to A (C to T); grey: A to C (T to G) or C to A (G to T); yellow: G to C or C to G.

WGS of 101 Thoroughbred racehorses revealed 11,570,312 and 602,756 SNVs from autosomal (1–31) and X chromosomes, respectively, in a total of 12,173,068 SNVs. One SNV was detected every 198 bp (= total base pairs of all chromosomes/12,173,068 SNVs) on average. The number of SNVs detected in individual horses ranged from 4.8 to 5.3 million (Table 1). The number of SNVs detected in the Thoroughbred population was lower than that detected in 88 horses from 25 diverse breeds (23,559,582 SNVs)^21^. The number of SNVs detected in each chromosome tended to be proportional to the chromosome length (Fig. 1).

Within the detected SNVs, the nucleotide substitutions with the highest frequency were A to G (T to C) or G to A (C to T), followed by A to C (T to G) or C to A (G to T), then G to C or C to G, and finally A to T or T to A. These mutation trends were common among all chromosomes (Fig. 1). Transition mutations that change a purine base (A and G) to another purine or a pyrimidine base (T and C) to another pyrimidine may occur most frequently. This tendency has also been observed in other species^22,23^.

### SNV density by genomic functional region

The SNV density (numbers of detected variants in each chromosomes whose size multiplied by scale factor 1000) was examined in genomic regions with different functions (intergenic, upstream and downstream regions, exon, intron, and untranslated region [UTR]) (Supplementary Table S2). Intergenic regions showed the highest density of SNVs (5.64745 [ECA12] to 2.25902 [ECA11]), followed by introns (2.96971 [ECA12] to 1.49113 [ECA26]), upstream (1.14895 [ECA12] to 0.22300 [ECA9]), downstream (1.07785 [ECA12] to 0.21970 [ECA9]), exon (0.04554 [ECA12] to 0.01441 [ECA4]), 3′-UTR (0.02638 [ECA12] to 0.00507 [ECA9]), and 5′-UTR (0.01727 [ECA12] to 0.00348 [ECA17]) in all chromosomes.

Both intergenic and intron regions are thought to have a high mutation rate because of the absence of gene coding regions. The variant rate in the intron was approximately half that in the intergenic region. This may be because some parts of introns are related to steps involved in the protein synthesis process, such as splicing, despite being non-coding for amino acids. Interestingly, the sequence of the UTR was more conserved than that of the exon region. One possible reason is that the UTR is involved in regulating expression by non-coding RNAs and RNA-binding proteins^24,25^.

**Figure 2:**
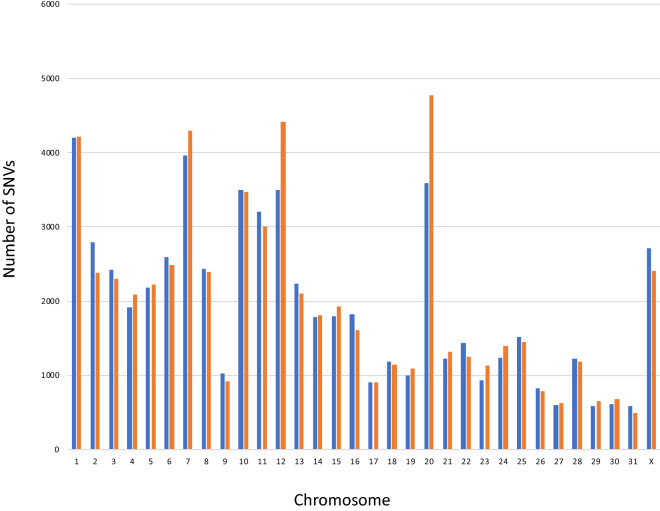
Distributions of synonymous and non-synonymous substitutions detected in gene-coding regions. Blue: synonymous; orange: non-synonymous.

No major differences were observed between the numbers of synonymous and non-synonymous substitutions detected in the gene-coding region (Fig. 2). However, the frequency of non-synonymous substitutions tended to be higher than that of synonymous substitutions in chromosomes 12 and 20, suggesting positive selection for higher variation in the amino acid sequence of proteins, such as those of the major histocompatibility complex^26,27^.

### Allelic distribution of detected SNVs

**Figure 3:**
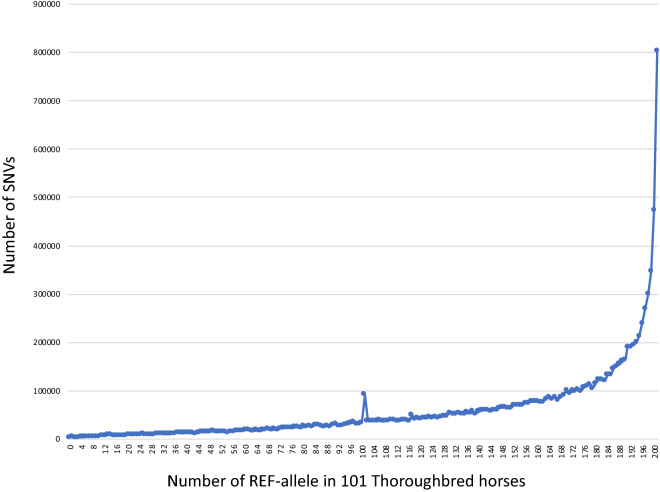
Allelic distributions of 11,570,312 SNVs identified on autosomes (ECA1-31) in the 101 Thoroughbreds. As can be seen on the right side, 802,454 SNVs (6.9%) had 201 REF-alleles and 1 ALT-allele in the 101 horses (total 202 alleles). A blip was observed at the position of 101 REF-alleles, which is equal to a minor allele frequency of 0.5.

The allelic distributions of 11,570,312 SNVs identified on autosomes (ECA1–31) were investigated in the 101 Thoroughbreds (Fig. 3). The right side of Fig. 3 shows the number of SNVs that had 201 REF-alleles and one ALT-allele in the 101 horses. The left side of Fig. 3 shows the number of SNVs that had 202 ALT-alleles among the 101 horses.

Interestingly, a blip was observed at the position of 101 REF-alleles, which corresponds to a minor allele frequency of 0.5. The horse reference genome sequence was produced using a Thoroughbred mare (Twilight)^18^, whereas genotypes of 3475 SNVs detected in the present study were homozygous for the alternative (ALT)-allele in the 101 horses (left side of Fig. 3), suggesting that Twilight may have unique SNVs that were not observed in our Thoroughbred population.

As can be seen on the right side of Fig. 3, 802,454 SNVs had only one ALT-allele among the 101 horses (total 202 alleles). Although depending on the definition of rare variants and/or individual numbers analysed, approximately 6.9% of the SNVs detected were rare variants in the Thoroughbred population. As we conducted WGS without PCR amplification, any bias in variant detection was expected to be low.

A similar trend was observed in variant detection using 88 horses from 25 breeds in horses^21^, and 12.5% of detected variants were specific to individual horses. A high frequency of rare variants was also observed in SNVs obtained from the 1000 Genomes Project in humans^28,29^. Interestingly, low-read depth WGS from 3,781 individuals of British ancestry identified over 42 million SNVs, approximately 80% of which were rare variants^30^. Therefore, by increasing the number of Thoroughbreds used for WGS, further rare variants may be identified.

### Application of common variants

Many SNVs were identified as common variants in this study. These SNVs may be useful as markers for Thoroughbred registration. Although short tandem repeats have been currently used internationally in parentage testing of Thoroughbreds, a test using SNVs has been considered by the International Society for Animal Genetics with 50 SNVs that we previously developed as candidates^31^. When searching the variant database for these candidates, polymorphic information can be easily extracted for all SNVs excluding one located at position 20379456 on ECA9 (Supplementary Table S3). Because reads mapped to the genome region containing the SNV on ECA9 showed low-mapping quality scores based on multiple-region mapping of reads, variant calling by GATK excluded this region. Therefore, SNVs on genomic regions with low-quality scores should not be used as markers for Thoroughbred registration. Additional SNVs for parentage verification in Thoroughbreds can be identified in our variant data.

### Duplicated regions in the horse genome

Analysis of the distribution of allele frequencies revealed a significant increase in the number of detected SNVs at the position of 101 ALT-alleles (Fig. 3). Although 91,835 SNVs on autosomal chromosomes (ECA1–31) had an MAF = 0.5, 61,788 (67.28%) of these were all-heterozygous genotypes (Supplementary Table S4). Interestingly, 13,402 of these SNVs were in the pericentromeric region (position: 1–2,293,071, approximately 2.3 Mb) of ECA29 (Supplementary Fig. S1), whereas the other SNVs were widely distributed in short lengths (several hundred base pairs). Analyses of individual mapping data using the Integrative Genomics Viewer and/or read depth values in VCF-files showed that the read depth (sequencing coverage) of the pericentromeric region was approximately twice that of the adjacent regions, and SNVs were distributed more densely in the pericentromeric region. The pericentromeric region of ECA29 may include duplicated sequences detected as pseudo-SNVs.

When analysing many individuals to confirm the genotype frequency, duplicated regions in the reference genome may be identified even in short read sequence data (150 bp). However, in the present study, we did not exclude SNVs with all-heterozygous genotypes from the total number of SNVs detected because we could not determine absolutely if they are duplication regions using only data from this study.

### Rare variants

**Figure 4:**
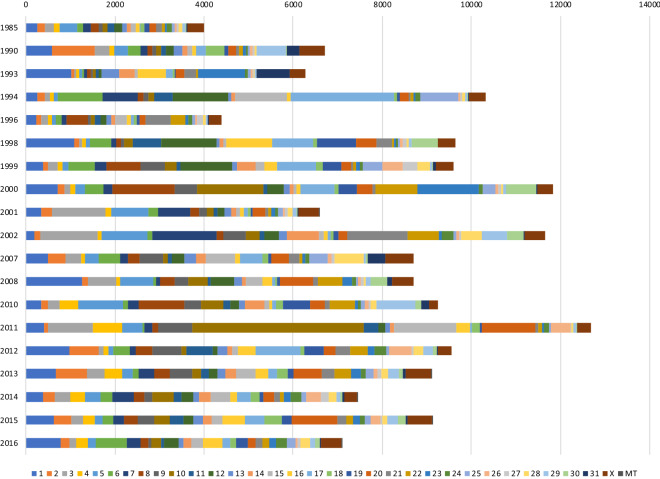
Mean number of rare variants detected from each horse grouped by birth-year. The number of horses born each year is shown in parentheses: 1985 (1 horse), 1990 (1), 1993 (1), 1994 (1), 1996 (1), 1998 (1), 1999 (3), 2000 (1), 2001 (1), 2002 (1), 2007 (5), 2008 (2), 2010 (2), 2011 (1), 2012 (4), 2013 (18), 2014 (20), 2015 (21), and 2016 (16). Colours represent different variant numbers on individual chromosomes.

In this study, we defined SNVs with only one ALT-allele detected among 101 horses as rare variants. When classified by birth year, the number of rare variants for earlier-born Thoroughbred individuals was lower than that for later-born individuals (Fig. 4), whereas horses born in later years tended to have higher numbers of rare variants. Horses born in 1985, 1990, 1993, and 1996 had 3994, 6713, 6279, and 4397 rare variants, respectively. Interestingly, these horses were used as stallions in Japan and had produced over 200 offspring. Particularly, the horse born in 1985 had over 500 offspring, several of which have become stallions and produced many progenies in Japan. In this case, it was considered that variants of the horse born in 1985 were inherited and increased in frequency within Japanese Thoroughbred horse populations, resulting in fewer rare variants.

Rare variants in individuals were characterised (Supplementary Table S5) and found to be present in coding region of genes as non-synonymous substitutions, start-gained, start-lost, stop-gained, and stop-lost, suggesting that the variants affect the phenotypes of each Thoroughbred horse as major and/or minor effects. Therefore, these functional rare variants in individual horses may explain the missing heritability based on the common disease/rare variant hypothesis^32^.

A variant identified in the horse myostatin gene was strongly associated with the optimal flat-racing distance in Thoroughbreds^4,5^. This variant may have been less frequent in the early Thoroughbred population^33^ and its frequency may have increased in current Thoroughbred populations by selective breeding through environmental adaptations (race distance), as short-distance races (1000 and 1200 m) have become more common over time. Therefore, some rare variants identified here may also contribute to racehorse performance and/or any traits in future populations by selective breeding through environmental adaptations.

### Allele frequencies of functional genes

Constructing a variant database in a Thoroughbred population facilitated easier identification of variants of annotated genes and their frequencies in the population. In horseracing, the generation and use of genetically modified horses are prohibited by the IFHA and International Stud Book Committee (ISBC). In our previous studies, we targeted 12 genes for gene-doping control in horseracing^13,15^. In the Thoroughbred population, we detected non-synonymous SNVs of some of the 12 genes in our variant database: 1 in *GH1*, 3 in *PCK1*, 1 in *VEGF*, and 2 in *ZFAT* (Table 2). This information may be useful for clarifying whether variants detected in doping tests are naturally or artificially introduced.

**Table 2:** Allelic frequencies of non-synonymous substitutions identified in candidate genes for gene-doping control.

Chr	Position	Gene	Allele	Amino acid	ALT-allele
			REF/ALT	REF/ALT	Freq
2	105,677,170	FGF2	C/A	Gln/Lys	0.0099
2	105,677,230	FGF2	G/A	Glu/Lys	0.1287
2	105,677,363	FGF2	T/C	Ile/Thr	0.0198
2	105,677,447	FGF2	G/C	Arg/Thr	0.0792
2	105,678,566	FGF2	T/C	Met/Thr	0.1436
9	77,071,461	ZFAT	T/C	Thr/Ala	0.2525
9	77,165,943	ZFAT	G/A	Thr/Met	0.1881
10	16,083,859	CKM	G/A	Val/Ile	0.0099
11	15,494,450	GH1	G/C	Gly/Ala	0.1188
20	43,602,385	VEGFA	A/C	Glu/Ala	0.0050
22	45,074,634	PCK1	G/A	Ala/Thr	0.3960
22	45,075,211	PCK1	C/G	Pro/Arg	0.0248
22	45,075,648	PCK1	A/G	Met/Val	0.2079

*Chr* chromosome, *REF* reference, *ALT* alternative, *Freq* frequency.

The drug-metabolising enzymes *CYP1A1* and *CYP1A2* also had one and four non-synonymous SNVs, respectively. In humans, CYP1A2 metabolises many low-molecular-weight compounds including caffeine and lidocaine^34^, which are banned as doping substances. Although the substrates in horse CYP1A2 are not completely clear^35^, differences in drug metabolism ability among individuals may be affected by SNVs with non-synonymous substitutions.

GWAS have been performed to identify SNVs associated with target traits and identified causative loci in Thoroughbreds^3–5^. It may be difficult to identify causative variants by only GWAS because common variants are used; however, the variant information of annotated genes constructed in this study can help in this identification through genotype imputation^36^.

### Diversity of the mitochondrial genome

**Figure 5:**
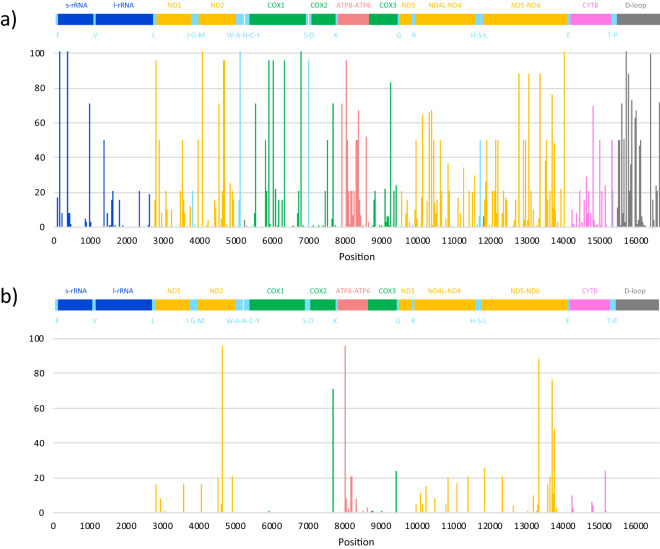
Numbers and locations of single-nucleotide variants (SNVs) detected in the mitochondrial genome. All detected (**a**) and non-synonymous substitutions (**b**) were plotted.

By mapping to the reference mitochondrial genome (16,660 bp)^37^, 335 SNVs were detected in 101 Thoroughbred horses (Fig. 5a). Seven of these SNVs occurred only as ALT-alleles and 58 of these SNVs were non-synonymous mutations among the 101 Thoroughbred horses (Fig. 5b).

Haplotypes of the mitochondrial genome were identified among the 101 horses and grouped by their founder mares based on Stud Book pedigree information. Interestingly, even if horses had the same founder mare in their female lines, the identified haplotypes clearly differed in several individuals. These discrepancies may result from errors in the original pedigree registration, and similar mistakes have been reported based on variations of mitochondrial genome^38^. In contrast, although the overall haplotype structures were consistent in the same founder mare, horses with a small number of mutations were also observed^39^. This may be because of natural mutations in their evolution over 300 years (approximately 30 generations) or miscalling of the identified variants.

### Concluding remarks

In this study, we constructed a genome-wide variant database from 101 Thoroughbred racehorses who did not have sibling or parent–child relationships. The number of detected SNVs may be overestimated because of miscalling from several duplicated regions, but our results revealed approximately 12 million SNVs among Thoroughbreds and that around 6.9% of these are rare variants.

A limitation of our dataset is the fact that current algorithms for mapping and variant calling will detect fake SNVs in duplicated regions in addition to genuine SNVs. Consequently, it is possible that our data could include up to tens of thousands of false-positive variant calls. Therefore, the improvement of algorithms for mapping and variant calling should be a future research priority.

The detected rare variants included many functional variants, such as non-synonymous variants, suggesting that rare functional variants affecting protein functions reflect individual phenotypes. In humans, rare variants are known to play a key role in many complex diseases, and rare variants in horses may also play a key role in Thoroughbred disease and/or racing performance.

The Equine Genetics and Thoroughbred Parentage Testing Standardization Committee of International Society for Animal Genetics is investigating the possibility of moving from short tandem repeats to SNVs as markers for determining parentage in Thoroughbreds. In the present study, we identified many SNVs as common variants and found duplicated regions in the horse genome and multiple mapped regions of sequence reads. Therefore, candidate SNVs for parentage verification should be selected from the common variants and exclude SNVs detected at these duplicated regions. The variant database in the present study can be used to confirm this.

Currently, the generation and use of genetically modified racehorses is banned by the ISBC and IFHA in horseracing. In the present study, we targeted only Thoroughbreds and determined the extent of Thoroughbred genomic diversity among the population of racehorses. Our findings will be useful as baseline information for gene-doping tests that use whole-genome and targeted resequencing.

## Materials and methods

### Animal ethics

All the experimental protocols were approved by the Animal Care Committee of the Laboratory of Racing Chemistry (approval number: 20-4) and was performed in accordance with the ARRIVE (Animal Research: Reporting of In Vivo Experiments) guidelines. The blood samples were collected from individual horses, the Hidaka Training and Research Center of the Japan Racing Association, and the Japan Bloodhorse Breeders’ Association, with permission for sample collection and research-use obtained from all owners.

### Animal samples and DNA extraction

Whole blood samples from 101 horses (58 males, 43 females) born between 1985 and 2016 were collected into BD Vacutainer^®^ spray-coated K2EDTA tubes (BD Biosciences, Franklin Lakes, NJ, USA). Genomic DNA was extracted from whole blood (200 μL) using a DNeasy Blood & Tissue Kit (Qiagen, Hilden, Germany). Extracted DNA was quantified using the Qubit dsDNA HS Assay Kit (Thermo Fisher Scientific, Waltham, MA, USA). Genomic DNA was diluted to 40 ng/μL using Milli-Q water (Merck, Kenilworth, NJ, USA).

### Whole-genome sequencing

Genomic libraries (550-bp insert size) for WGS were prepared using the TruSeq DNA PCR-Free Low Throughput Library Prep Kit (Illumina, San Diego, CA, USA) according to the manufacturer’s recommendations and were quantified by digital PCR with the Digital PCR Library Quantification Kit (Bio-Rad, Hercules, CA, USA). Sequencing was carried out in-house on a NextSeq 500 sequencing platform (Illumina) using NextSeq 500/550 v2.5 Kits (Illumina), or by Macrogen Japan Corp. (Koto, Tokyo, Japan) using NovaSeq 6000 and HiSeq X sequencing platforms.

### SNV calling and filtering

SNV detection was carried out using the RESEQ pipeline (Amelieff Co., Minato, Tokyo, Japan), which was constructed using QCleaner (Amelieff Co.), Burrows-Wheeler Aligner (version 0.7.17), Picard (version 2.13.2) (https://sourceforge.net/apps/mediawiki/picard/), GATK HaplotypeCaller (version 4.0.8.1) (https://software.broadinstitute.org/gatk/best-practices/), and SnpEff (version v4_0). The SnpEff annotated gene information for EquCab3.0 was derived from the reference FASTA and GTF files.

Briefly, using QCleaner, reads with a low-quality base (< 20 Phred score) were removed. The remaining reads were filtered and removed if 80% of their nucleotides had a quality value < 20, had sequences of over five unknown nucleotides, had only < 32 bp length sequences, or did not have mate-pairs. Only high-quality sequences were selected.

The selected reads were aligned to the horse reference genome sequence EquCab3.0 assembly from GenBank (GCA_002863925.1) using Burrows-Wheeler aligner with default parameters. Alignments were converted from sequence alignment/map format to sorted, indexed binary alignment/map files (SAMtools, version 1.8), and then the Picard tool was used to remove duplicate reads. Finally, binary alignment/map files were constructed with mapping data to the horse reference genome.

GATK was used to detect SNVs with default parameters. The SNVs were filtered using the GATK VariantFiltration program with the following criteria: clusterWindowSize:10, MQ0 ≥ 4& ((MQ0/(1.0*DP)) > 0.1), DP < 10, QUAL < 50, QD < 1.5, SB > -0.1. SNVs were annotated using SnpEff. Finally, VCF files were constructed with variant data for resequenced horses.

### Database construction and statistical analyses

Information from VCF files for individual horses, chromosome, position, reference allele, alternative allele, gene, HGVSp, annotation, and annotation impact was collected into a single file using the Vcf2sql (Amelieff Co.), from which allele frequencies and SNP density (numbers of detected variants in each chromosomes whose size multiplied by scale factor 1000) were calculated.

## Supplementary Information

Supplementary Figure S1.

Supplementary Table S1.

Supplementary Table S2.

Supplementary Table S3.

Supplementary Table S4.

Supplementary Table S5.

## Data Availability

Variant data in 101 Thoroughbred can be accessed through the Open Science Framework, 10.17605/OSF.IO/PVNCY.
