# Supplementary material for: Rare and common variant discovery by whole-genome sequencing of 101 Thoroughbred racehorses
Source: Sci Rep. 2021 Aug 6;11:16057. doi: 10.1038/s41598-021-95669-1 (PMC8346562; doi:10.1038/s41598-021-95669-1)
Supplement: Supplementary file 1 — Supplementary Figure S1. [file 41598_2021_95669_MOESM1_ESM.pdf]

Rare and common variant discovery by whole-genome sequencing of 101 Thoroughbred racehorses

Teruaki Tozaki, Aoi Ohnuma, Mio Kikuchi, Taichiro Ishige, Hironaga Kakoi, Kei-ichi Hirora, Kanichi Kusano, Shun-ichi Nagata

Supplementary information

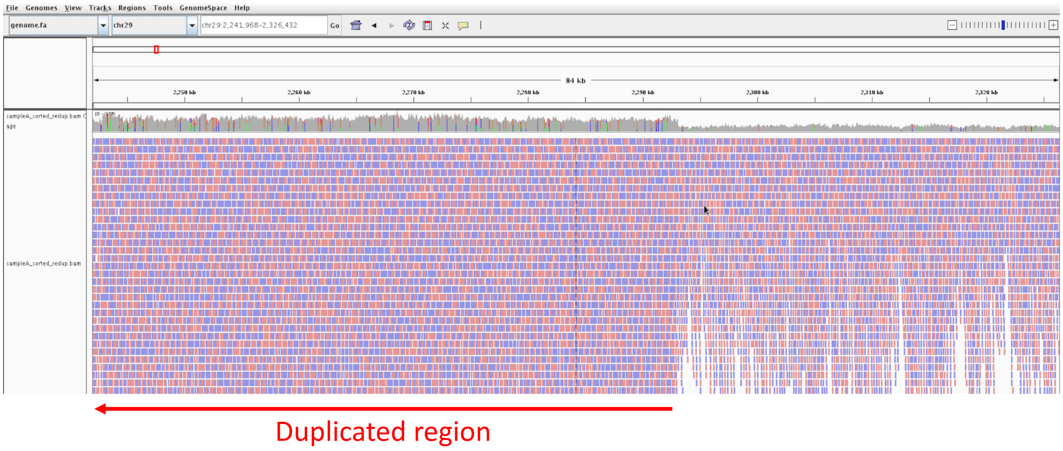

Figure S1. Duplicated regions detected by genotype frequency distributions.
A pericentromeric region on ECA29 (1-2,293,071: approximately 2.3 Mb) was deeply mapped compared with the adjacent region and had a high density of single nucleotide variants (SNVs).
